# Inferring within-patient HIV-1 evolutionary dynamics under anti-HIV therapy using serial virus samples with vSPA

**DOI:** 10.1186/1471-2105-10-360

**Published:** 2009-10-29

**Authors:** Naoki Hasegawa, Wataru Sugiura, Junko Shibata, Masakazu Matsuda, Fengrong Ren, Hiroshi Tanaka

**Affiliations:** 1 School of Biomedical Sciences, Medical and Dental University, Yushima 1-5-45, Bunkyo, Tokyo 113-8510, Japan; 2 Center for Information Medicine, Medical and Dental University, Yushima 1-5-45, Bunkyo, Tokyo 113-8510, Japan; 3 AIDS Research Center, National Institute of Infectious Diseases, Gakuen 4-7-1, Musashi-Murayama, 208-0011, Tokyo, Japan; 4 Clinical Research Center, National Nagoya Medical Center, Sanno-Maru 4-1-1, Naka, Nagoya, Aichi 460-0001, Japan; 5 Mitsubishi Chemical Medicine Corporation, Shimura 3-30-1, Itabashi, Tokyo 174-8555, Japan

## Abstract

**Background:**

Analysis of within-patient HIV evolution under anti-HIV therapy is crucial to a better understanding the possible mechanisms of HIV drug-resistance acquisition. The high evolutionary rate of HIV allows us to trace its evolutionary process in real time by analyzing virus samples serially collected from the same patient. However, such studies are still uncommon due to the lack of powerful computational methods designed for serial virus samples. In this study, we develop a computational method, vSPA (viral Sequential Pathway Analysis), which groups viral sequences from the same sampling time into clusters and traces the evolution between clusters over sampling times. The method makes use of information of different sampling times and traces the evolution of important amino acid mutations. Second, a permutation test at the codon level is conducted to determine the threshold of the correlation coefficient for clustering viral quasispecies. We applied vSPA to four large data sets of HIV-1 protease and reverse transcriptase genes serially collected from two AIDS patients undergoing anti-HIV therapy over several years.

**Results:**

The results show that vSPA can trace within-patient HIV evolution by detecting many amino acid changes, including important drug-resistant mutations, and by classifying different viral quasispecies coexisting during different periods of the therapy.

**Conclusion:**

Given that many new anti-HIV drugs will be available in the near future, vSPA may be useful for quickly providing information on the acquisition of HIV drug-resistant mutations by monitoring the within-patient HIV evolution under anti-HIV therapy as a computational approach.

## Background

HIV is one of the fastest evolving viruses ever known. Its high evolutionary rate is the main reason for rapid acquisition of drug resistance and the difficulty of vaccine development. However, the fast rate can also be utilized in evolutionary analysis, enabling us to trace the evolutionary process of HIV in real time by analyzing virus samples serially collected from infected individuals.

Many previous studies have examined HIV population dynamics, and revealed many unique and important features of viral evolution within an infected individual [1-6]. Numerous viral variants coexist in the same host which arises from the rapid genomic evolution powered by the high mutation rate during viral replication. Some of these studies described the viral population by using the concept of "quasispecies", a dynamic distribution of non-identical but closely related mutants, which acts as a unit of selection [7-12]. Therefore, in viral evolution, the target of selection is not the individual mutant sequence. When the patient is under Highly Active Antiretroviral Therapy (HAART), which has been executed on AIDS patients since the early 1990s, the greater selective pressure on the viral population is probably the anti-HIV drugs. The multiple viral groups with different drug-resistant mutations coexisting within a single patient will have different potentials to respond to the internal and external selective pressures. Moreover, whenever the HAART regime is changed, new mutations on the targeted genes emerge rapidly at sites related to drug resistance, enabling the virus to escape from drug attacks, while most sites of the viral gene remain almost entirely unchanged over a relatively short period. Understanding this complex process is crucial for elucidating the possible mechanisms of the anti-HIV drug resistance acquisition, which has become a major challenge in anti-HIV therapy.

In contrast to the rapid accumulation of viral data, few powerful methods for inferring the within-patient viral evolution are available. One reason appears to be the difficulty to deal with serial virus samples [13,14]. Traditional phylogenetic methods such as neighbor-joining (NJ) [15] and maximum likelihood (ML) [16] are designed for contemporaneous molecular data and do not take into account sampling times. Drummond and Rodrigo [17] proposed the sUPGMA method, which extends conventional UPGMA to serially sampled data. Like other methods for serial data [18,19], sUPGMA considers all viral variants as the end points of evolution and ignores the fact that in serial samples, viral variants survive or leave descendents to the next time point. The same problem exists in the Bayesian method of Drummond et al. [20]. A further difficulty is that the viral sequences from the same patient are extremely similar and thus uninformative about their phylogenetic relationships, so that phylogeny-based methods suffer from uncertainties in the reconstructed phylogenies. Recently, Buendia and Narasimhan [14] developed the Sliding MinPD method, which reconstructs the evolutionary networks of serial viral sequences by combining minimum pairwise distances with automated recombination detection based on a sliding window approach. This method overcomes some of the limitations of traditional phylogenetic tree reconstruction and is able to detect recombination events, which may occur frequently during HIV replication. The method is not widely used and its performance is not well-understood. Furthermore, Beerenwinkel and Drton [21] developed a mutagenetic tree model to describe the order of occurrences of amino acid changes that are associated with resistance to a particular drug. The method assumes that we know which amino acid mutations are important to drug resistance.

We propose a new method, vSPA (viral Sequential Pathway Analysis), for analyzing within-patient HIV evolution. We calculate the genetic distances between virus samples, and use Pearson correlation coefficient to classify viral variants at the same sampling time into different clusters. We further identify the evolutionary pathways between viral clusters across the sampling times. A permutation test at the codon level is used to determine the threshold correlation coefficient for the clustering. We applied the vSPA method to four large data sets of HIV-1 protease (PR) and reverse transcriptase (RT) genes serially collected from two Japanese AIDS patients, and compared the results with those from traditional tree-building methods and Sliding MinPD.

## Results

### Viral clusters and evolutionary pathways inferred by vSPA

The vSPA method was applied to four large data sets of PR and RT genes serially collected from Patients 1 and 2. Below, we refer to these data sets as P1PR, P1RT, P2PR and P2RT, respectively.

#### Mutually exclusive mutations observed in P1PR

The results of the permutation test for P1PR are shown in Additional file 1 and the distributions of correlation coefficients for the randomly generated data differed considerately from those for real HIV-1 data.

**Figure 1:**
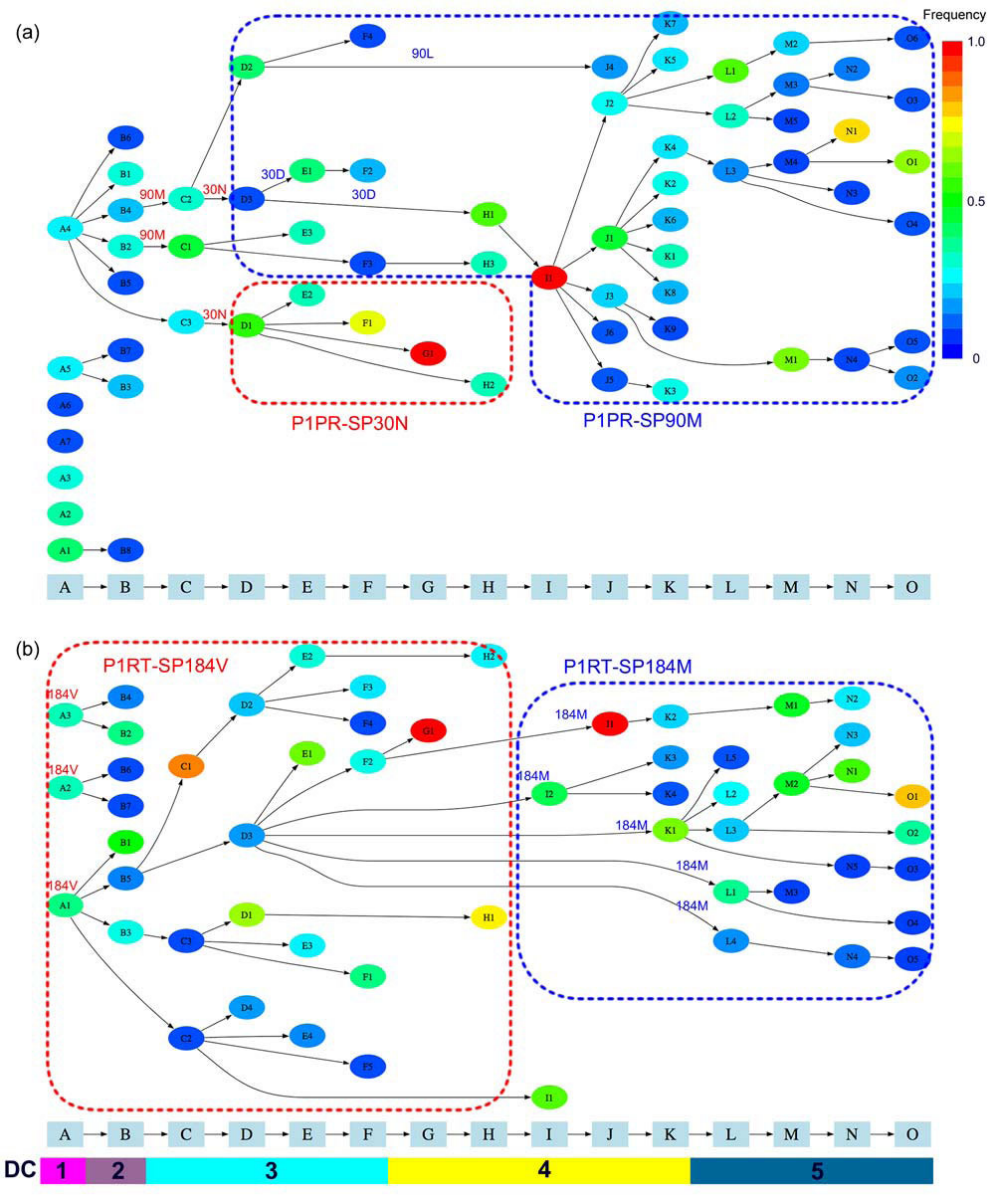
**Evolutionary pathways of 273 PR genes (a) and 287 RT genes (b) from Patient 1 inferred using vSPA**. Sampling time points A, B, ..., O are indicated on the horizontal axis, as well as the drug combination (DC) executed. The ellipses represent viral clusters inferred by the vSPA method. The clusters are ordered by the number of sequences they contain and are colored according to their frequencies. For example, at time point A, A1 has the most sequences among the clusters A, A2, ..., A7. Black arrows indicate the inferred evolutionary pathway. Important drug-resistant mutations are indicated on the pathway and the subpopulations divided according to the drug-resistant mutation are enclosed with dotted lines.

**Figure 2:**
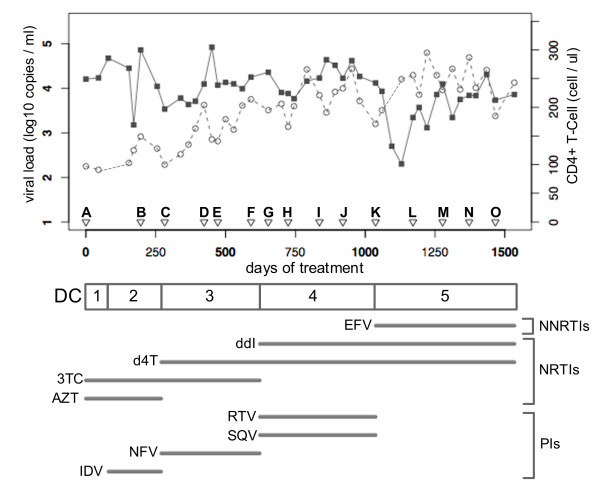
**Anti-HIV treatment history of Patient 1**. Viral load (black square) and CD4+ T-cell counts (grey circle) are plotted against the days of anti-HIV treatment. A, B, ... O indicate sampling time points. DC (Drug Combinations) 1, 2, ... 5 represent five periods, respectively, in which different drug combinations were administered, with the drugs used indicated below. NNRTIs: non-nucleotide reverse transcriptase inhibitors; NRTIs: nucleotide reverse transcriptase inhibitors; PIs: protease inhibitors.

The clusters and their evolutionary dynamics inference for the 273 P1PR sequences are shown in Figure 1a. Viral diversity fluctuated over the observation period. At time points A and B, many clusters existed, but very few clusters were constructed for time points C-I. Only one cluster was estimated for time points G and I. However, the virus diversified again from time point H. The diversity changes corresponded to the change in the protease inhibitors (PIs) administered to Patient 1 (see Figure 2).

From the amino acid mutations that each cluster possessed (see Additional file 2), it appeared most likely that two subpopulations existed with different frequencies during different periods. One is a subpopulation possessing D30N drug-resistant mutation, which we refer to as P1PR-SP30N, and the other a subpopulation possessing L90 M drug-resistant mutation, which we refer to as P1PR-SP90 M. The P1PR-SP30N was observed from time point D to H and was a main subpopulation until time point G. However, it declined in frequency at time point H and disappeared at time point I. The emergence and extinction of this subpopulation apparently corresponded with the administration of the Nelfinavir (NFV), to which the D30N is a major drug-resistance mutation. In contrast, the P1PR-SP90 M emerged at time point C and existed over all later time points. It had been a minor subpopulation until time point G and became major from time point H--that is, 103 days after NFV was switched to Ritonavir (RTV) and Saquinavir (SQV). The L90 M is a well-known drug-resistant mutation for multiple PIs. The above result was consistent with the experimental finding that the D30N and L90 M drug-resistant mutations were mutually exclusive [22].

#### M184V had the greatest impact on P1RT evolution

The evolutionary dynamics of P1RT is depicted in Figure 1b. Two subpopulations, P1RT-SP184V and P1RT-SP184 M, probably existed in the earlier and latter periods, respectively. The mutation M184V has a strong drug resistance to Lamivudine (3TC) and P1RT-SP184V was the major subpopulation from time points A to I, but was replaced completely by the subpopulation having the wild-type 184 M at time point J about 7 months after the switch from 3TC to Dinanosine (ddI). This result agrees with the experimental observation that M184V occurs in a small proportion of patients but does not occur in patients receiving ddI with Zidovudine (ZDV) or Stavudine (d4T) [23].

#### Possible convergent evolution in P2PR

**Figure 3:**
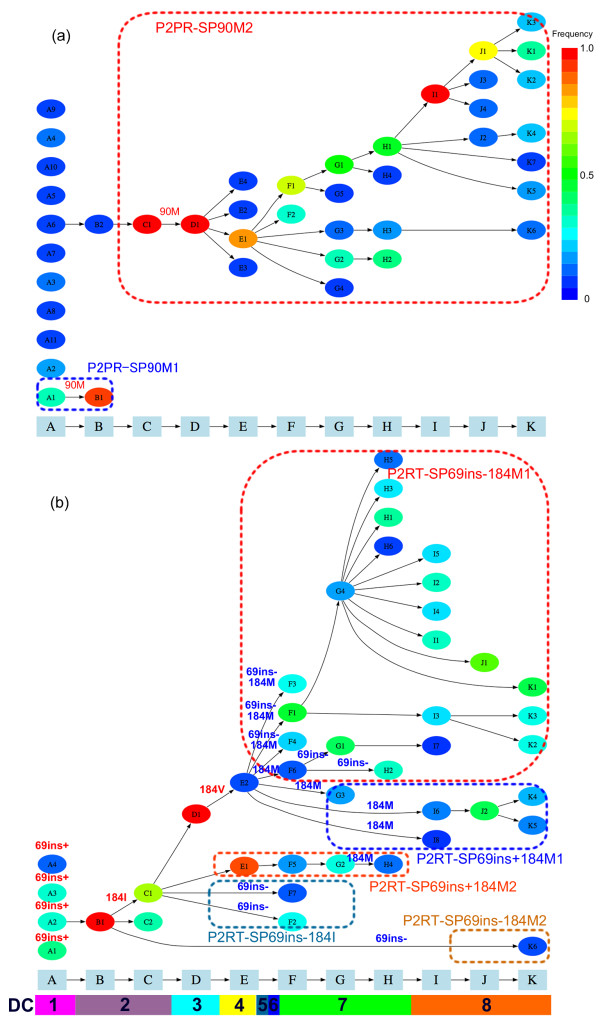
**Evolutionary pathways of 240 PR genes (a) and 207 RT genes (b) from Patient 2 inferred using vSPA**. Sampling time points A, B, ..., K are indicated on the horizontal axis. See the legend to Figure 1 for details. Note that ins- means absence of the T69 insertion and ins+ presence.

**Figure 4:**
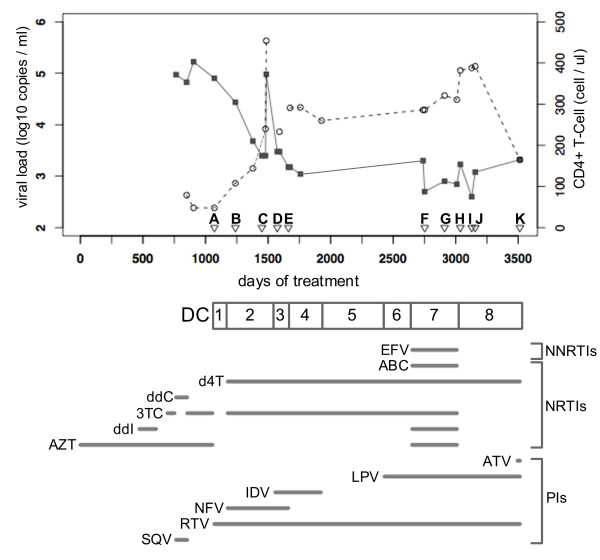
**Anti-HIV treatment history of Patient 2**. Viral load (black square) and CD4+ T-cell counts (grey circle) are plotted against the days of anti-HIV treatment. A, B, ... K indicate sampling time points. DC (Drug Combinations) 1, 2, ... 8 represent eight periods, respectively, in which different drug combinations were administered, with the drugs used indicated below. NNRTIs: non-nucleotide reverse transcriptase inhibitors; NRTIs: nucleotide reverse transcriptase inhibitors; PIs: protease inhibitors.

The result obtained from analyzing the 240 samples of P2PR are shown in Figure 3a. The diversity pattern was different from that of Patient 1. For time point B to E, only one or two clusters were constructed. As shown in Figure 4, the PIs had been administered before the sampling and a multiple drug-resistant mutation, L90 M, was observed since time point A. This subpopulation, however, could not survive longer, and another subpopulation emerged after time point C instead. Because both subpopulations possessed the mutation L90 M, we called them P2PR-SP90 M1 and P2PR-SP90 M2. By comparing clusters B1 with C1 and D1 at the codon level (Table 1), we observed more common codons in clusters C1 and D1, thus it is most likely that the 90 M mutation occurred by convergent evolution in two subpopulations. However, a common codon (TGC) was observed at site 95 in clusters B1 and D1 but not in cluster C1 (TGT), raising a possibility that cluster D1 might be recombinant between clusters B1 and C1 as well.

**Table 1:** Different amino acids and codons in clusters B1, C1 and D1 of P1PR

**Position**	**Cluster**		
	
	**B1**	**C1**	**D1**
10	L(CTC)	**I(ATC)**	**I (ATC)**
32	V(GTA)	**V(GTG)**	**V (GTG)**
35	E(GAA)	**D (GAC)**	**D (GAC)**
37	**D(GAT)**	**D (GAT)**	E (GAA)
54	I(ATC)	**T (ACC)**	**T (ACC)**
55	R(AGA)	**K (AAA)**	**K (AAA)**
62	**I(ATA)**	**I (ATA)**	V (GTA)
71	A(GCT)	***V (GTT)***	***V (GTG)***
90	**M(ATG)**	L (TTG)	**M (ATG)**
95	**C(TGC)**	C (TGT)	**C (TGC)**

Identical codons in any two clusters are shown in bold. Identical amino acids at different codon levels are shown in bold italics.

#### Co-evolution observed in P2RT

The results for 207 samples of P2RT are shown in Figure 3b. We focused on two evolutionary events, T69 insertion and M184VI mutation. They are two major drug-resistant mutations to d4T and ddI and to 3TC, respectively. We further classified the viral clusters into 5 subpopulations for the period of high diversity from time point E: SP69ins-184 M1, SP69ins-184 M2, SP69ins+184 M1, SP69ins+184 M2 and SP69ins-184I. Note that ins- means absence of the T69 insertion and ins+ presence.

The T69 insertion was observed at time point A. The presence of this insertion is probably related to its resistance to several nucleoside RT inhibitors (NRTIs) that had been administered to Patient 2 before the sampling started. The M184VI mutation occurred between time points B and E. The five subpopulations were mainly observed from time point F, before which no sample was taken for more than three years. Briefly, the two subpopulations with T69 insertion and back mutation at site 184 (SP69+184 M1 and SP69+184 M2) were observed, but they were not dominant except at time point E. The SP69ins-184I had been dominant for only a short period as well. In contrast, the SP69ins-184 M1, which lost T69 insertion and acquired back mutation at site 184 in the same period, diversified and had been a major subpopulation for a long period. This observation raises a possibility that there might be interaction between the lost T69 insertion and the back mutation at site 184.

The SP69ins-184 M2 subpopulation included only one sequence k5 and had neither T69 insertion nor M184V mutation, but it was linked with a very early cluster B1. We investigated the difference between the clusters of time point K and found that only sequence k5 possessed the M230L mutation, a drug-resistant mutation to Enfuvirtide (EFV). Also, another mutation, K103R, was observed in the k5 alone. It is likely that sequence k5 was a memory variant having resistant mutations to EFV, given that this drug had not been administered for more than one year.

All mutations existing at the first time point and newly acquired on the pathways can be found in Additional file 2.

### Comparison with phylogenetic reconstruction methods and MinPD

We applied two representative methods, Neighbor-joining (NJ) [15] and maximum likelihood (ML) [16], to the same data sets to reconstruct phylogenetic trees, using MEGA4 [24] and PHYML [25], respectively. The K80 + G model [26,27] was used as for vSPA. Bootstrap analysis with 1000 replicates was performed on the reconstructed phylogenetic trees.

The NJ and ML methods produced very similar trees (see Additional file 3). First, the bootstrap values were very low at almost all internal nodes on all trees. Second, as the sampling times were not taken into account in either NJ or ML, the inferred trees showed unreasonable structures for evolutionary relationships, with virus samples obtained at later time points to be descendents of variants obtained earlier.

The same data sets were also analyzed using the Sliding MinPD method [14]. Considering the different lengths of the two genes, we set the window size to be 100 bp for PR and 200 bp for RT. The default offset (20 bp) was used in all analyses. The results for the P1PR dataset are shown in Additional file 4, while those for the three other datasets are not shown because of their large file sizes. Many recombination events were inferred by Sliding MinPD for the P1PR, P1RT, and P2RT datasets, making it very difficult to find evolutionary pathways from these networks.

## Discussion

In this study, we developed a new method for analyzing serial virus samples and applied it to the large data sets of PR and RT genes of HIV-1 obtained from two AIDS patients undergoing HAART. Here we discuss some strengths and weaknesses of the vSPA method, in comparison with conventional tree-reconstruction methods and the Sliding MinPD method.

Traditional tree-building methods were found to be unsuitable for the analysis of serial virus samples. Not only did they confuse ancestor-descendant relationships due to their failure to account for sampling times but the reconstructed tree topologies also had poor accuracy due to the high similarities of viral sequences. Similarly, the networks constructed by the Sliding MinPD method might not be useful to reveal evolutionary relationships among viral variants, as the method appeared to detect too many recombination events.

In contrast, the vSPA method has several advantages for analyzing serial virus samples with high similarity. First, the ancestor-descendent relationships among viral sequences are accommodated by the method, as different sampling times are taken into account. Our method also accounts for latent viral variants as we searched for ancestors over all previous time points during pathway construction. vSPA infers viral ancestral relationships at the cluster level and appeared to be more useful for interpreting the evolutionary relationships among viral sequences than methods that attempt to infer relationships at the individual sequence level. Second, viral variants inferred to be in the same cluster not only have high sequence similarities but also share important evolutionary features such as drug-resistant mutations. Our results suggest that multiple viral groups with different mutations usually coexist within the same patient during different periods and their frequencies fluctuate in response to changes in drug combinations. While fluctuation of viral mutations in within-host evolution is noted before [5,28-30], the vSPA method analyzes this process quantitatively.

Third, vSPA traces the acquisition, frequency change, and loss of important drug-resistant mutations over time (see Additional file 2), and classifies the viral subpopulations according to these amino acid changes. In this regard, it has advantages over other cluster methods. For example, PAQ (Partition Analysis of Quasispecies), developed by Baccam et al. (2001), clusters viral variants based on their genetic diversity, but requires the user to specify a radius value for clustering. For the data analyzed in this paper, the genetic similarities are so high that it is difficult to select a sensible radius value. In contrast, vSPA uses a permutation test to determine the threshold correlation coefficient automatically. Our permutation test is conducted at the codon level to test interactions among different codons of the HIV genome - such interactions may be important for the PR and RT genes, which are the targets of the main anti-HIV drugs at present.

A number of amino acid changes were inferred in both genes for both patients, although only a few examples were discussed due to limited space. We note that many of the amino acid changes identified by vSPA were already known to be responsible for resistance to anti-HIV drugs. Our analysis also identified some previously unknown amino acid changes, which may provide interesting hypothesis for experimental verification.

The vSPA method has weaknesses as well. It does not automatically detect recombination although it is possible to infer recombination events by confirming those common amino acid changes in some clusters (see Table 1). One strategy may be to use standard recombination-detection methods (e.g., RIP etc.) to detect recombination on the viral clusters inferred by vSPA. A problem with almost all recombination detection methods is that they ignore natural selection and are known to generate excessive false positives when the gene is under strong selection. The PR and RT genes analyzed in this study are collected from the AIDS patients under anti-HIV therapy and they are main targets of anti-HIV drugs. No doubt they are under strong positive selection which drives drug-resistant mutations to fixation. As a result, we lack reliable estimates of recombination rates for those data. Analytical methods that deal with recombination and selection simultaneously are still in their infancy [31]. We believe that recombination should have less impact on our method, which is based on pairwise distance calculation, than on phylogeny-based methods. Nevertheless, the effect of recombination on vSPA remains unknown and merits further research.

## Conclusion

Since many new anti-HIV drugs will be available in the near future and more complex drug-resistant mutations will emerge, vSPA may be useful for analyzing viral responses to those drugs and for providing valuable information to experimental researchers quickly. We hope that our study will motivate the development of computational methods suitable for viral quasispecies that take into account major features of the data, such as different sampling times, and dramatic changes in selective pressure due to changing drug combinations.

## Methods

### Subjects and viral sequences

Two subjects (Patients 1 and 2) were selected for this study. Patient 1 is a 46-year-old male with hemophilia. His treatment history and clinical course were monitored by National Institute of Infectious Diseases (NIID) in Tokyo from 1996 to 2001 (Figure 2). Patient 2 is a 26-year-old male and was infected with HIV-1 by blood products. He has been administered anti-HIV drugs since 1994 and his treatment history and clinical course were monitored by NIID from 2004 (Figure 4).

All viral data analyzed in this study were sampled and sequenced at the AIDS Research Center of NIID. The details of the cloning and sequencing can be found in previous papers [32,33]. From Patient 1, 273 samples of the PR gene (297 bp) and 287 samples of the RT gene (787 bp) were obtained at 15 time points, with 15-25 samples at each time point. For Patient 2, 240 samples of the PR gene (297 bp) and 207 samples of the RT gene (787 bp) were obtained from 10 time points, with 15-25 samples for each time point. The sequences were aligned using ClustalW, followed by manual alignment.

### vSPA algorithm

#### Creating viral clusters based on the correlation coefficient of genetic distances

The clustering procedure in vSPA involves several steps. The first step is to create viral clusters for time point *T*_*n*_, *n *= 1, 2, ..., *N*, where *N *is the number of sampling time point. A pairwise distance matrices *D*_*n *_is calculated under the K80+G [26] model for sequences of time point *T*_*n *_and the previous time point *T*_*n*-1_.

(1)

where *l*_*n *_and *l*_*n*-1 _are the number of sequences at time points *T*_*n *_and *T*_*n*-1_, respectively. Note that, for the first time point, no earlier variants exist. The K80 + G model [26] with pairwise deletion was used for the calculation of genetic distance in this analysis, implemented using the R package APE. The proportions of the four nucleotides were inferred from viral samples. Other substitution models can be chosen in vSPA as well. As the sequences are very similar, different substitution models were noted to produce nearly identical distance estimates.

Second, we normalized *D*_*n *_and calculated matrix *Z*_*n *_as follows.

(2)

Here, *z*_*ij *_is the element in the matrix *Z*_*n *_and is given as

(3)

Where the mean value  and variance  for each row of the matrix *D*_*n *_are calculated as

(4)

(5)

Third, we used the normalized matrix to calculate the correlation coefficient matrix *R*_*n*_.

(6)

For *n *= 1, we replaced *l*_*n*-1 _+ *l*_*n *_with *l*_*n*_.

**Figure 5:**
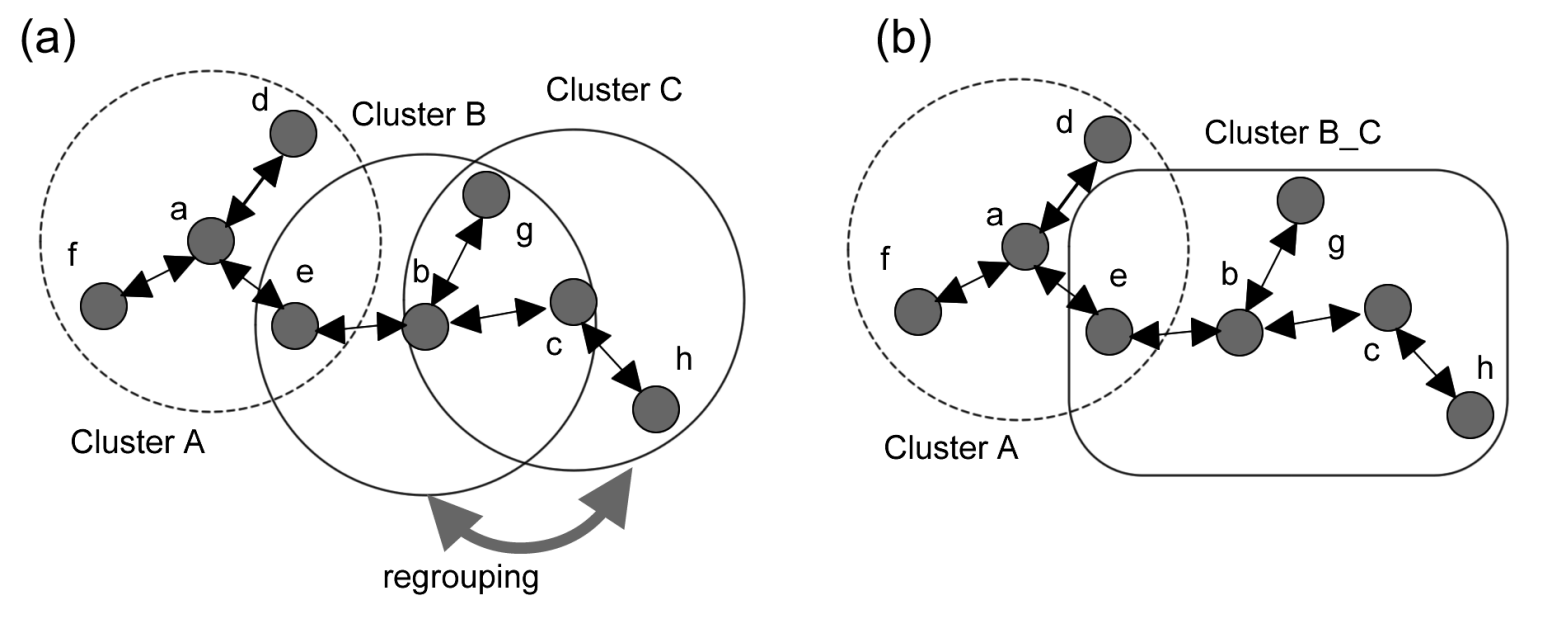
**Clustering procedure of vSPA**. (a) Illustration of the clustering procedure in vSPA. The big circles indicate the clusters classified using the cutoff value of the correlation coefficient, whereas the small solid circles represent individual variants, distinguished by lower-case letters. (b) The big square represents the cluster formed by merging clusters that share more than 50% of viral variants.

Two variants whose correlation exceeds a threshold (see below) are grouped into the same cluster. Some variants could be grouped into more than one cluster because of the high similarity of the viral sequences; thus, clusters sharing more than half of their variants were merged to form a larger cluster (Figure 5). The merging was repeated until the number of clusters no longer changed.

#### Conducting permutation test

As the diversity of viral variants depends on the sampling time point, it may not be appropriate to use the same correlation coefficient threshold for all time points to cluster viral variants. We implemented a permutation test to determine the correlation coefficient to be used for each time point. Sequences in a viral cluster not only are highly similar but also possess characteristic drug-resistant mutations. To present the coding structure and to accommodate the co-occurring and interesting amino acid mutations, we conduct the permutation test at the codon level.

**Figure 6:**
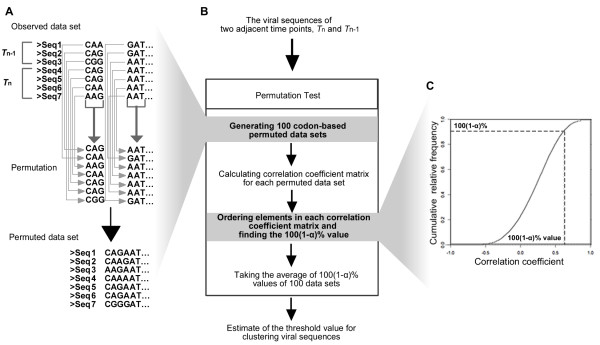
**Outline of the permutation test conducted in vSPA**. α = 0.1 is used in this study.

First, the sequences at two adjacent time points, *T*_*n *_and *T*_*n*-1_, were used in the permutation, with each codon rearranged randomly among the viral sequences to generate the data at the codon in the permutation data set (Figure 6A). This procedure was repeated to generate 100 permutation data sets. Second, for each permutation data set we calculated the correlation coefficient matrix, and find the 100(1 - α)% value when the correlation coefficients in the matrix are ranked, with α = 0.1 used in this study (Figure 6B &6C). Finally, we took the average of 100(1 - α)% values among the 100 permutation data sets as the threshold correlation coefficient for clustering *T*_*n *_viral variants. The procedure here not only resamples the viral sequences, but also disturbs the evolutionary interactions among codons.

#### Identifying ancestral viruses

In vSPA, the ancestor-descendant relationships were inferred at the cluster level and all variants grouped into the same cluster were assumed to have a common ancestor (cluster) in one of the previous time points. Since the ancestor of viral variants at time point *T*_*n *_may not be sampled at time point *T*_*n*-1_, we traced the evolutionary pathways to all time points before *T*_*n *_to find the ancestor for each cluster of time point *T*_*n*_. We took the average of the genetic distances between each *T*_*n *_cluster and each *T*_*n*-*m *_(*m *= 1, 2, 3) cluster over all the viral variants, and then chose the *T*_*n*-*m *_cluster with the minimum average distance as the ancestor of the *T*_*n *_cluster. When only a few sequences are included in a cluster, the calculated average distance may involve large sampling errors. However, this procedure of linking clusters of sequences appears to be more adequate for clustering highly similar sequences than linking sequences of shortest distances between clusters, as we do not know which single viral clone can be used as the representative of a cluster. By repeating this procedure, the ancestral cluster of each *T*_*n *_cluster was identified. Then, we connected the ancestral clusters over the entire observation period to construct the evolutionary pathway. The graph-drawing tool Graphviz (version 10) was used to visualize the pathways and to output the results of vSPA automatically.

## Availability of data and programs

Four datasets analyzed in this study and the programs developed to carry out the vSPA algorithm are included in Additional file 5. The vSPA package is available at .

## Authors' contributions

NH, HT and FR conceived and designed the method. NH wrote the program and analyzed the data. FR performed the MinPD. WS, JS and MM carried out the viral experiments. FR and NH drafted the manuscript. All authors read and approved the final manuscript.

## Supplementary Material

Additional file 1 **The results of permutation test of P1PR**. This file includes 15 figures which show the results of permutation test for each sampling time point of P1PR. Click here for file

Additional file 2 **Acquired mutations inferred from PR and RT genes of Patients 1 and 2**. This file includes four tables in which all mutations inferred by vSPA from P1PR, P1RT, P2PR and P2RT are listed. Click here for file

Additional file 3 **Reconstructed phylogenetic trees by using the NJ and ML methods**. The eight phylogenetic trees reconstructed from P1PR, P1RT, P2PR and P2RT are shown in this file. Click here for file

Additional file 4 **The evolutionary network constructed using Sliding MinPD method**. This file shows the evolutionary network constructed from the 273 PR genes of Patient 1 by Sliding MinPD. Click here for file

Additional file 5 **The vSPA programs and four datasets analyzed in this study**. The programs developed to carry out the vSPA algorithm and four datasets analyzed in this study and are included in this ZIP file. Click here for file
