# Supplementary material for: Inferring within-patient HIV-1 evolutionary dynamics under anti-HIV therapy using serial virus samples with vSPA
Source: BMC Bioinformatics. 2009 Oct 29;10:360. doi: 10.1186/1471-2105-10-360 (PMC2776027; doi:10.1186/1471-2105-10-360)
Supplement: Additional file 1 — The results of permutation test of P1PR. This file includes 15 figures which show the results of permutation test for each sampling time point of P1PR. [file 1471-2105-10-360-S1.pdf]

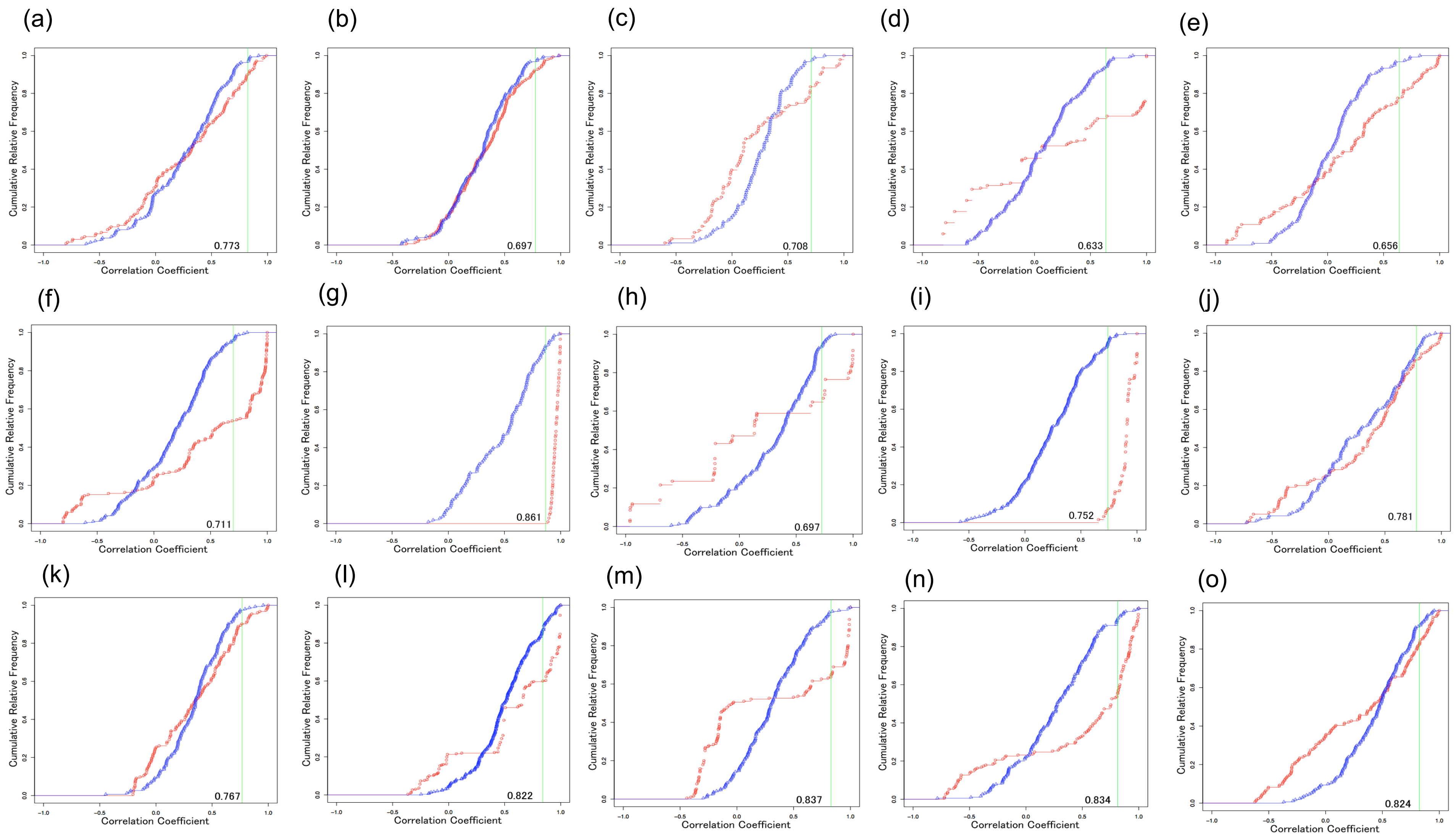

The results of permutation test of P1PR. (a) ~ (o) are for sampling time points A-O in Figure 1. The horizontal axis indicates the correlation coefficient and the vertical axis indicates the cumulative relative frequency. The red circles represent the observed viral data while the blue circles indicate the randomized data by permutation.
