# Supplementary material for: Inferring within-patient HIV-1 evolutionary dynamics under anti-HIV therapy using serial virus samples with vSPA
Source: BMC Bioinformatics. 2009 Oct 29;10:360. doi: 10.1186/1471-2105-10-360 (PMC2776027; doi:10.1186/1471-2105-10-360)
Supplement: Additional file 2 — Acquired mutations inferred from PR and RT genes of Patients 1 and 2. This file includes four tables in which all mutations inferred by vSPA from P1PR, P1RT, P2PR and P2RT are listed. [file 1471-2105-10-360-S2.doc]

**Mutations acquired along pathways estimated from P1PR**

| *Root cluster* | *Mutation* | *Category* |
| --- | --- | --- |
| A1 | L10R | Accessory mutation |
|  | I15V | Unknown |
|  | E34D | Unknown |
|  | K43K | Back mutation |
|  | M46L | PI resistant mutation |
|  | I62V | Accessory mutation |
| A4 | L10V | Accessory mutation |
|  | L10I | Accessory mutation |
|  | V11I | Accessory mutation |
|  | K14K | Back mutation |
|  | I15V | Unknown |
|  | G16E | Unknown |
|  | K20T | Unknown |
|  | A22V | Unknown |
|  | D30N | PI resistant mutation |
|  | L38V | Back mutation |
|  | R41R | Back mutation |
|  | K43K | Back mutation |
|  | K45N | Unknown |
|  | M46I | PI resistant mutation |
|  | M46L | PI resistant mutation |
|  | M46V | Unknown |
|  | G49E | Unknown |
|  | K55R | Accessory mutation |
|  | I62V | Accessory mutation |
|  | I72I | Back mutation |
|  | G73S | PI resistant mutation |
|  | T74S | Accessory mutation |
|  | V77I | Accessory mutation |
|  | I84V | PI resistant mutation |
|  | N88D | PI resistant mutation |
|  | L89P | Unknown |
|  | L90M | PI resistant mutation |
| A5 | T12T | Back mutation |

Root cluster: estimated as the root of evolutionary pathways by vSPA.

Mutation: existing at the first time point and newly acquired on the pathways

Category: classified based on HIV Drug Resistance Database of Stanford University.

**Mutations acquired along pathways estimated from** P1RT

| Root cluster | Mutation | Category | Root cluster | Mutation | Category |
| --- | --- | --- | --- | --- | --- |
| A1 | K13R | Unknown | A1 | P133S | Unknown |
|  | M16V | Unknown |  | E169K | Unknown |
|  | E28K | Unknown |  | F171L | Unknown |
|  | E29K | Unknown |  | K173K | Back mutation |
|  | C38R | Unknown |  | Q174K | Unknown |
|  | K43N | NRTI other mutation |  | M184M | Back mutation |
|  | E44K | Unknown |  | G190S | NNRTI resistant mutation |
|  | K49K | Back mutation |  | T200A | Unknown |
|  | V60I | Unknown |  | E203K | Unknown |
|  | D67N | TAMs |  | L210L | Back mutation |
|  | T69D | NRTI other mutation |  | R211K | Unknown |
|  | K70R | TAMs |  | H221Y | NRTI other mutation |
|  | L74V | non-TA NAMs | A2 | I31T | Unknown |
|  | V75A | NRTI other mutation |  | V35T | Unknown |
|  | P95S | Unknown |  | K43R | Unknown |
|  | H96R | Unknown |  | T69D | NRTI other mutation |
|  | L100I | NNRTI resistant mutation |  | K73E | Unknown |
|  | K101P | NNRTI resistant mutation |  | D113G | Unknown |
|  | K101E | NNRTI resistant mutation |  | S156P | Unknown |
|  | K101Q | NNRTI other mutation |  | Q197R | Unknown |
|  | K102R | Unknown |  | Q197K | Unknown |
|  | K103N | NNRTI resistant mutation |  |  |  |
|  | V118I | NRTI other mutation |  |  |  |
|  | E122E | Back mutation |  |  |  |

**Mutations acquired along pathways estimated from** P2PR

| Root cluster | Mutation | Category |
| --- | --- | --- |
| A1 | G48V | PI resistant mutation |
|  | K55R | Accessory mutation |
|  | V82A | PI resistant mutation |
|  | L90M | PI resistant mutation |
| A6 | L10I | Accessory mutation |
|  | I13V | Accessory mutation |
|  | I15I | Back mutation |
|  | K20R | Accessory mutation |
|  | A22V | Unknown |
|  | L33F | PI resistant mutation |
|  | E35D | Unknown |
|  | M36L | Unknown |
|  | S37E | Unknown |
|  | K43T | Accessory mutation |
|  | G48V | PI resistant mutation |
|  | F53L | Unknown |
|  | I54S | Other mutation |
|  | I54T | PI resistant mutation |
|  | I54V | PI resistant mutation |
|  | D60E | Accessory mutation |
|  | I62V | Accessory mutation |
|  | I64I | Back mutation |
|  | A71I | Accessory mutation |
|  | A71V | Accessory mutation |
|  | I72V | Unknown |
|  | G73S | PI resistant mutation |
|  | V77I | Accessory mutation |
|  | V82A | PI resistant mutation |
|  | I85V | Accessory mutation |
|  | L90M | PI resistant mutation |
|  | I93L | Accessory mutation |

**Mutations acquired along pathways estimated from P2RT**

| Root cluster | Mutation | Category |
| --- | --- | --- |
| A2 | P4S | Unknown |
|  | E29G | Unknown |
|  | V35I | Unknown |
|  | M41M | Back mutation |
|  | E42K | Unknown |
|  | K43E | NRTI other mutation |
|  | E44K | Unknown |
|  | A62V | Multi-NRTI resistant mutation |
|  | 69ins[AS] | Multi-NRTI resistant mutation |
|  | Delete 69ins[SS] | Back mutation |
|  | T69T | Back mutation |
|  | T69G | NRTI other mutation |
|  | T84A | Unknown |
|  | W88S | Unknown |
|  | K101R | NNRTI other mutation |
|  | V118I | NRTI other mutation |
|  | E122E | Back mutation |
|  | S134G | Unknown |
|  | I167V | Unknown |
|  | E169D | Unknown |
|  | M184I | non-TA NAMs |
|  | M184V | non-TA NAMs |
|  | L228P | Unknown |
