# Supplementary material for: Inferring within-patient HIV-1 evolutionary dynamics under anti-HIV therapy using serial virus samples with vSPA
Source: BMC Bioinformatics. 2009 Oct 29;10:360. doi: 10.1186/1471-2105-10-360 (PMC2776027; doi:10.1186/1471-2105-10-360)
Supplement: Additional file 3 — Reconstructed phylogenetic trees by using the NJ and ML methods. The eight phylogenetic trees reconstructed from P1PR, P1RT, P2PR and P2RT are shown in this file. [file 1471-2105-10-360-S3.pdf]

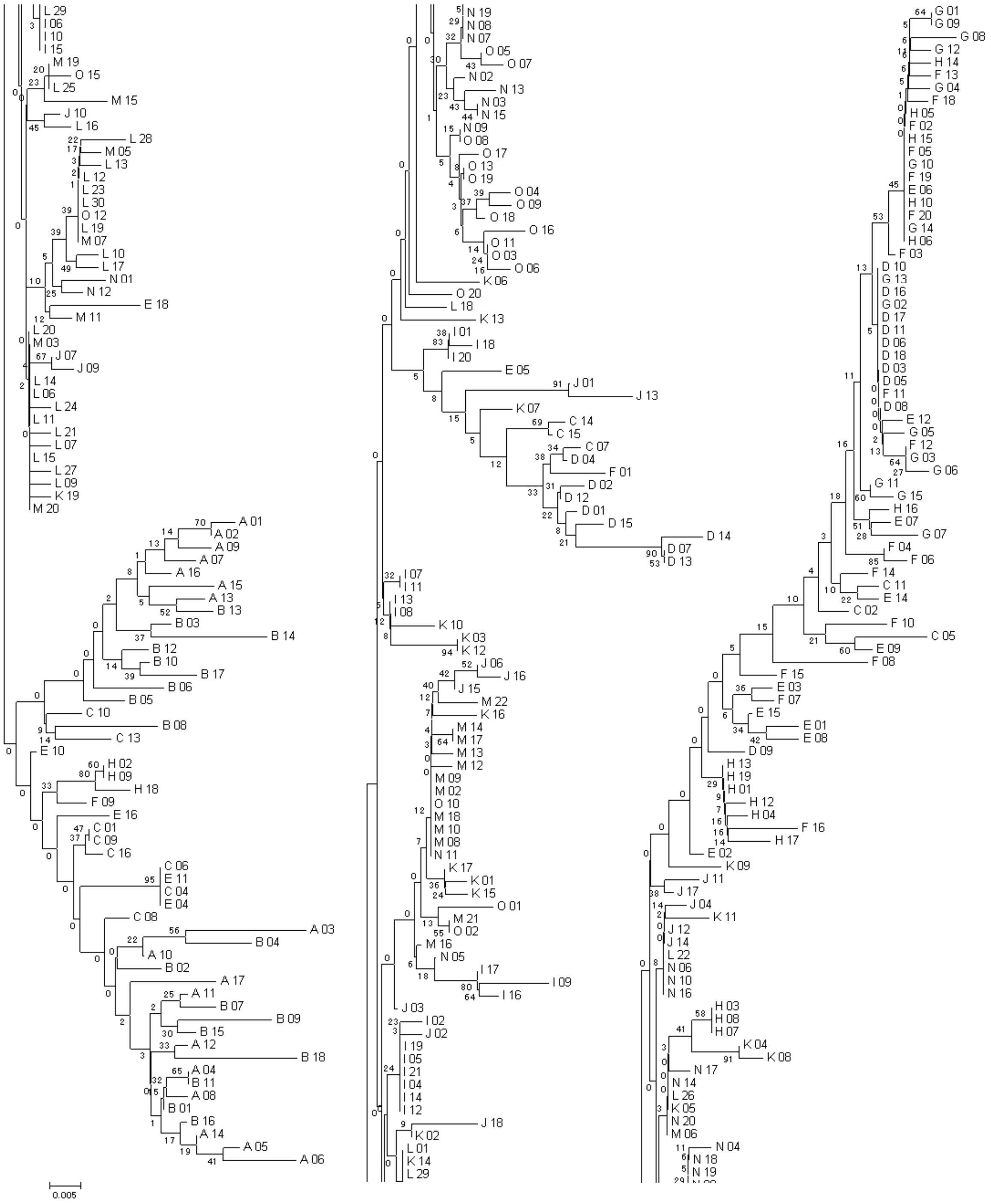

(a) Reconstructed phylogenetic tree of 273 protease genes from Patient 1 using the neighbor-joining method (MEGA4.0). Sequences from time point A are used as the root. The capital letters in the sequence name represent the time points (see Figure 1).

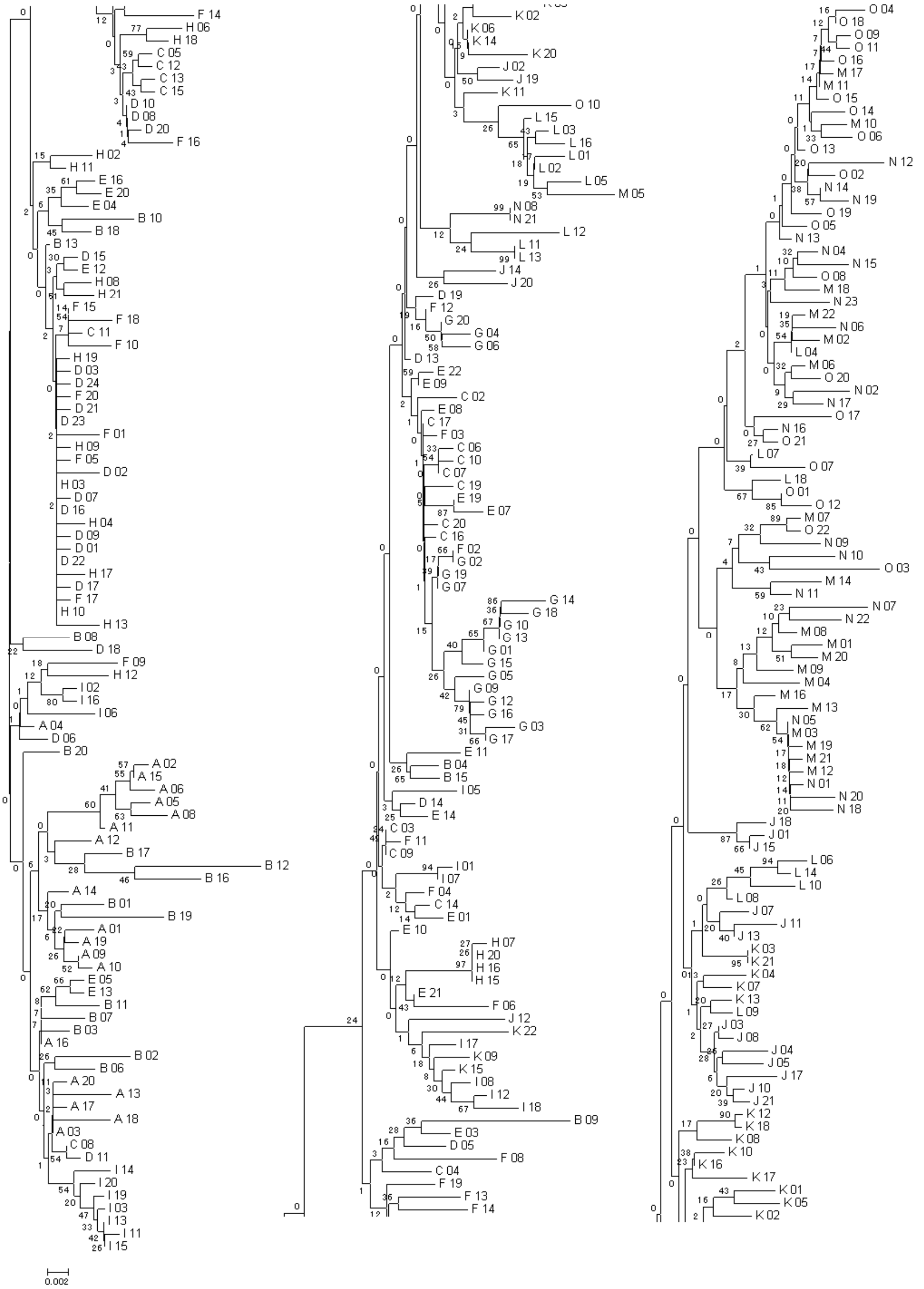

(b) Reconstructed phylogenetic tree of 287 reverse transcriptase genes from Patient 1 using the neighbor-joining method (MEGA4.0). See the legend to (a) for details.

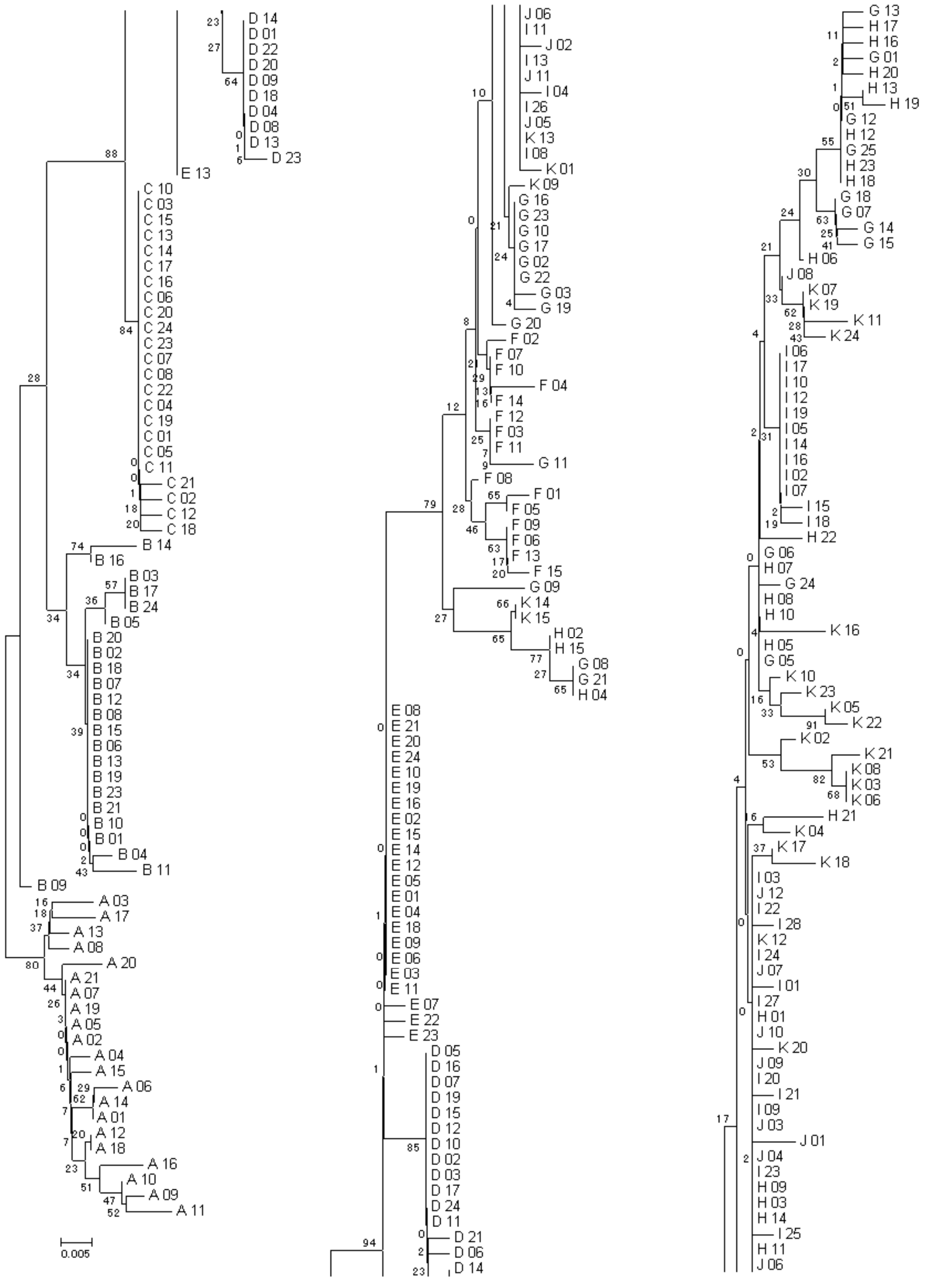

(c) Reconstructed phylogenetic tree of 240 protease genes from Patient 2 using the neighbor-joining method (MEGA4.0). Sequences from time point A are used as the root. The capital letters in the sequence name represent the time points (see Figure 2).

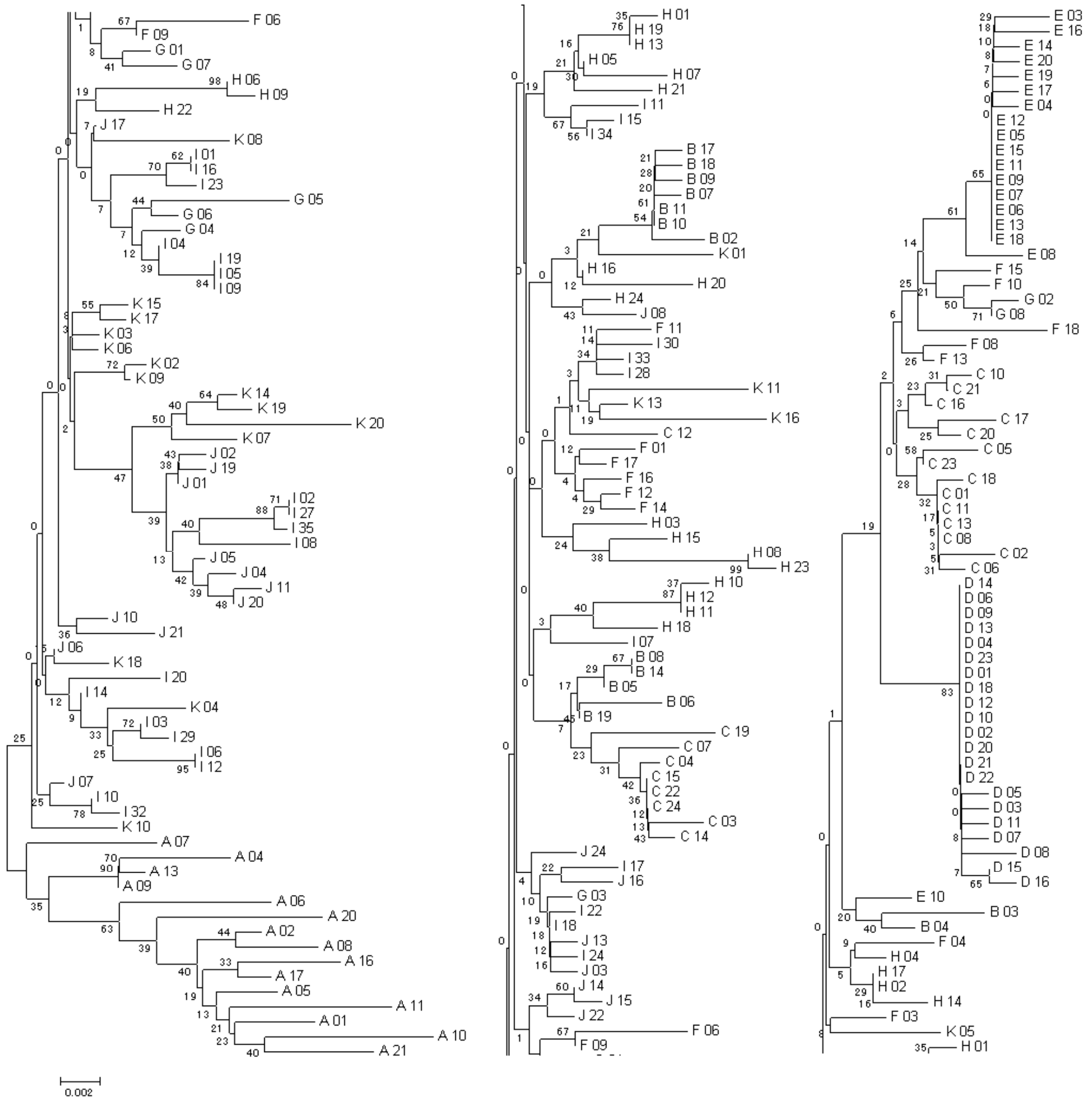

(d) Reconstructed phylogenetic tree of 207 reverse transcriptase genes from Patient 2 using the neighbor-joining method (MEGA4.0). See the legend to (c) for details.

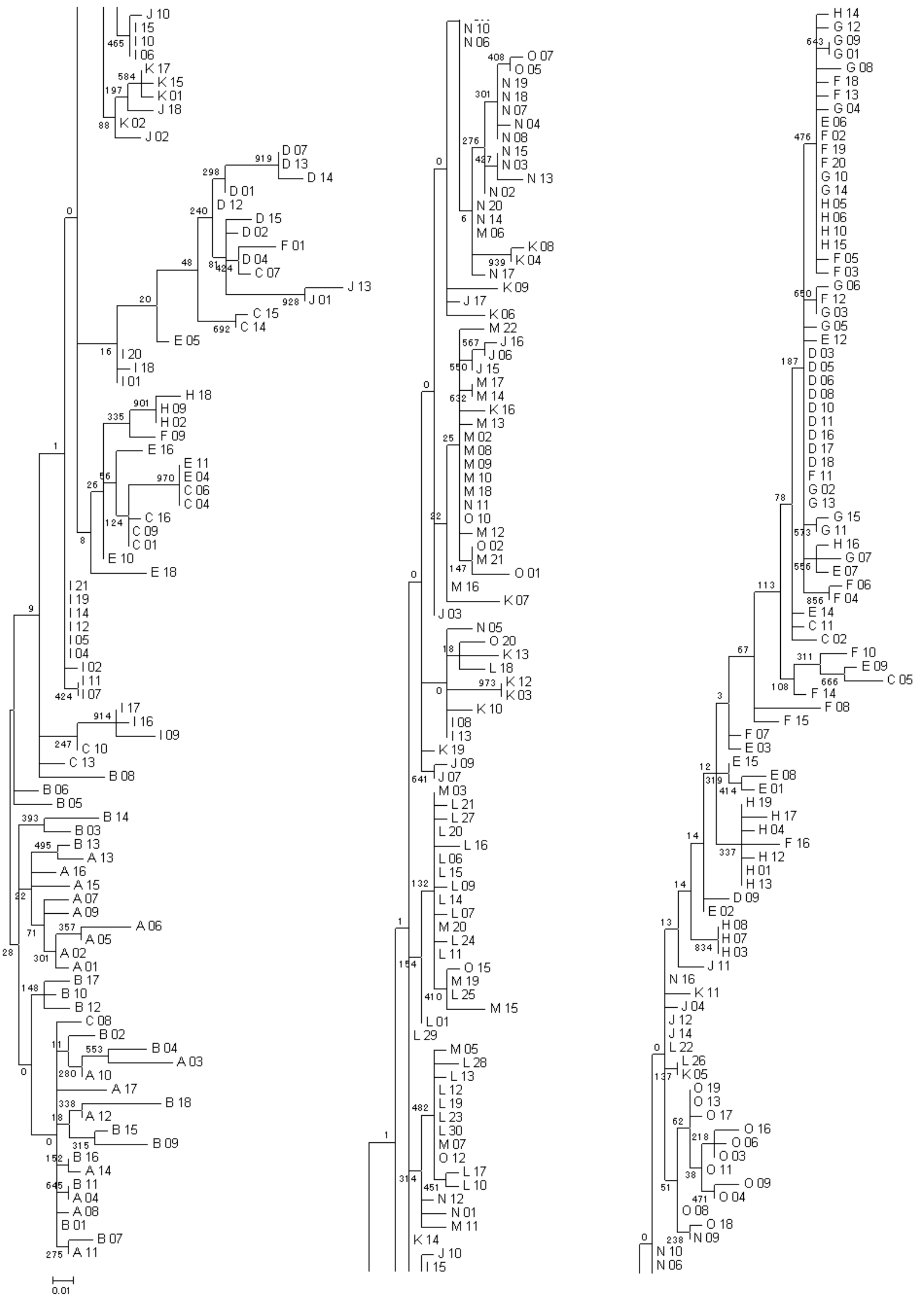

(e) Reconstructed phylogenetic tree of 273 protease genes from Patient 1 using the maximum likelihood method (PHYML). Sequences from time point A are used as the root. The capital letters in the sequence name represent the time points (see Figure 1).

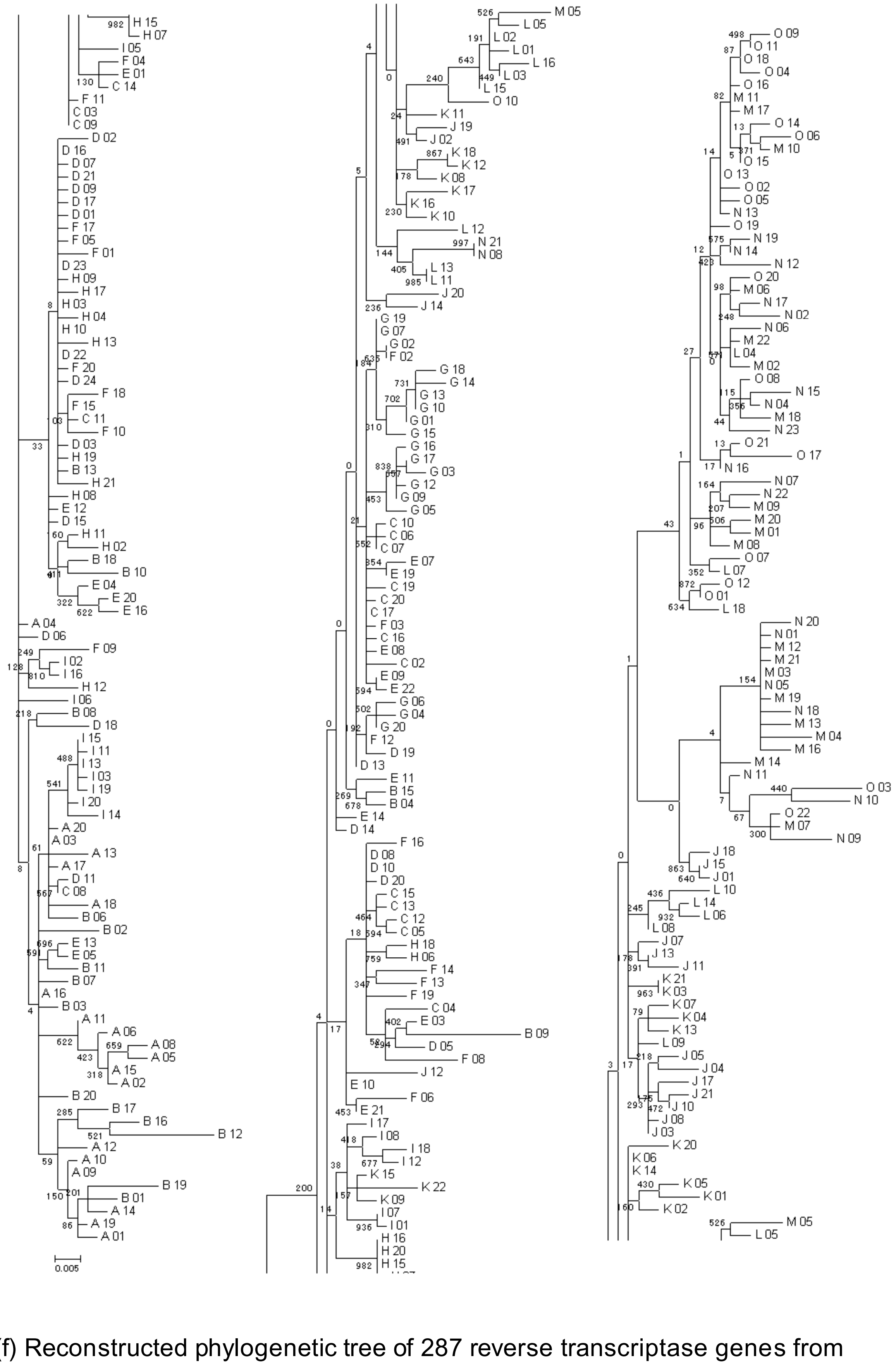

(f) Reconstructed phylogenetic tree of 287 reverse transcriptase genes from Patient 1 using the maximum likelihood method (PHYML). See the legend to (e) for details .

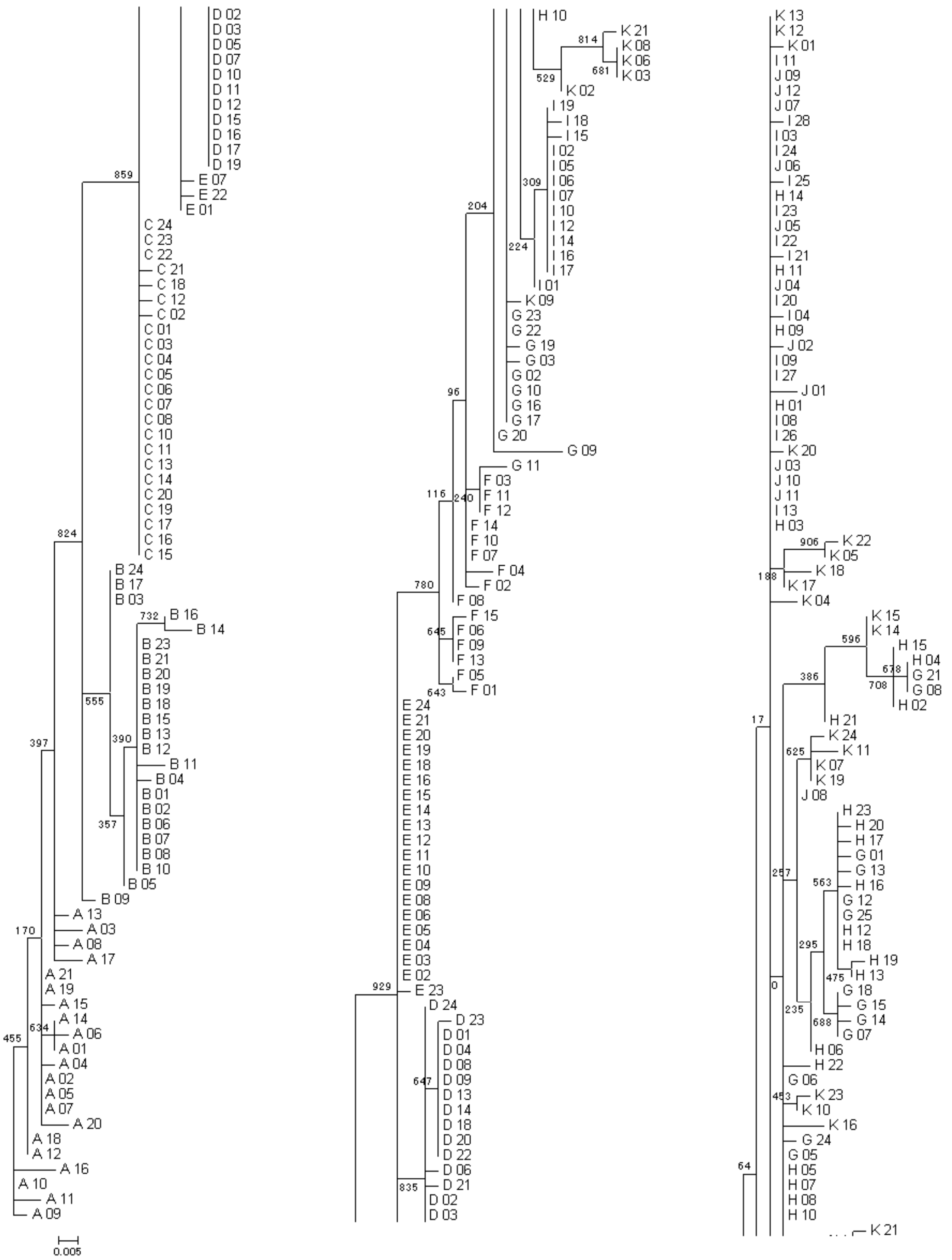

(g) Reconstructed phylogenetic tree of 287 reverse transcriptase genes from Patient 2 using the maximum likelihood method (PHYML). Sequences from time point A are used as the root. The capital letters in the sequence name represent the time points (see Figure 2).

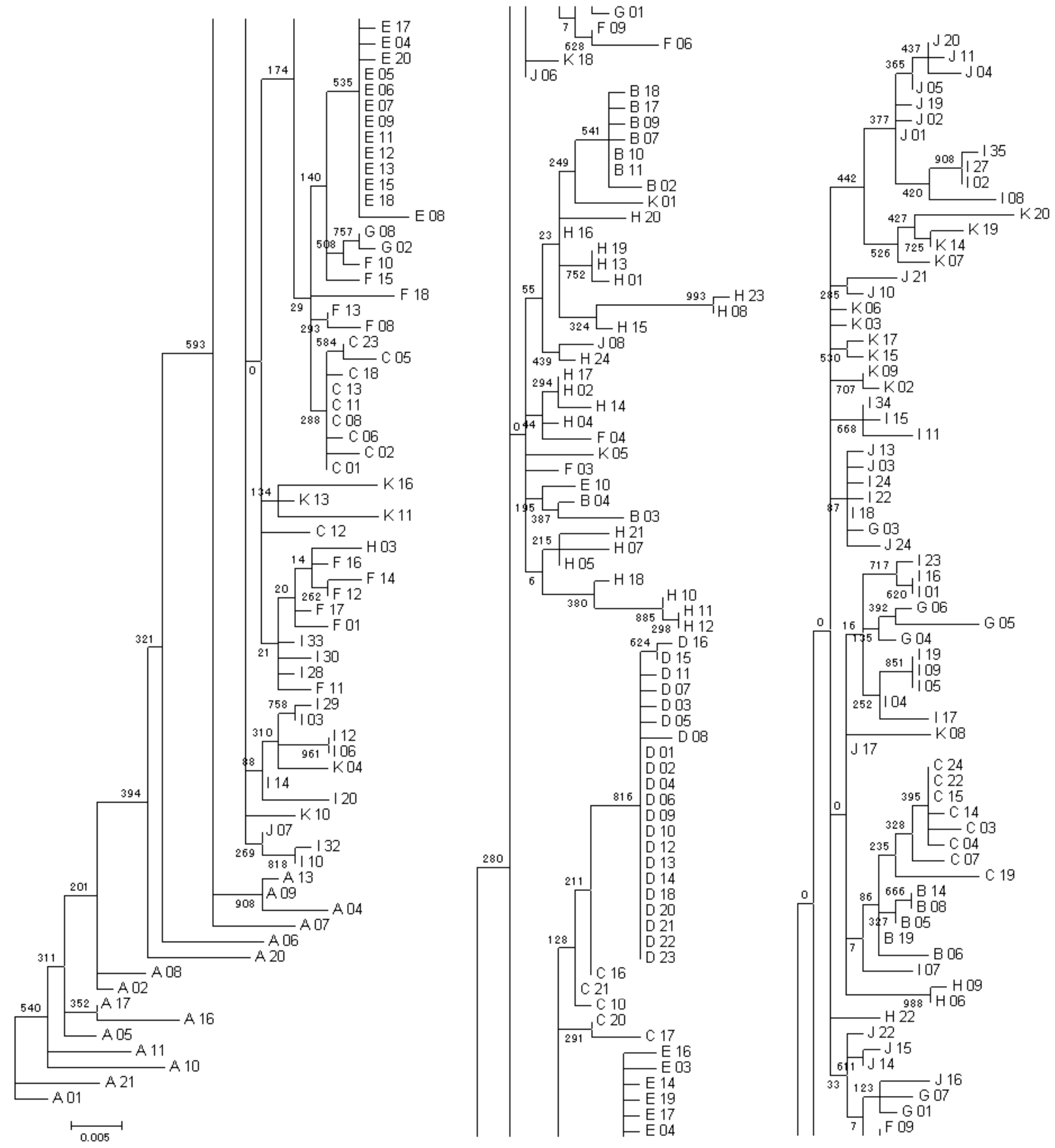

(h) Reconstructed phylogenetic tree of 287 reverse transcriptase genes from Patient 2 using the maximum likelihood method (PHYML). See the legend to (g) for details.
