# Supplementary material for: Inferring within-patient HIV-1 evolutionary dynamics under anti-HIV therapy using serial virus samples with vSPA
Source: BMC Bioinformatics. 2009 Oct 29;10:360. doi: 10.1186/1471-2105-10-360 (PMC2776027; doi:10.1186/1471-2105-10-360)
Supplement: Additional file 4 — The evolutionary network constructed using Sliding MinPD method. This file shows the evolutionary network constructed from the 273 PR genes of Patient 1 by Sliding MinPD. [file 1471-2105-10-360-S4.pdf]

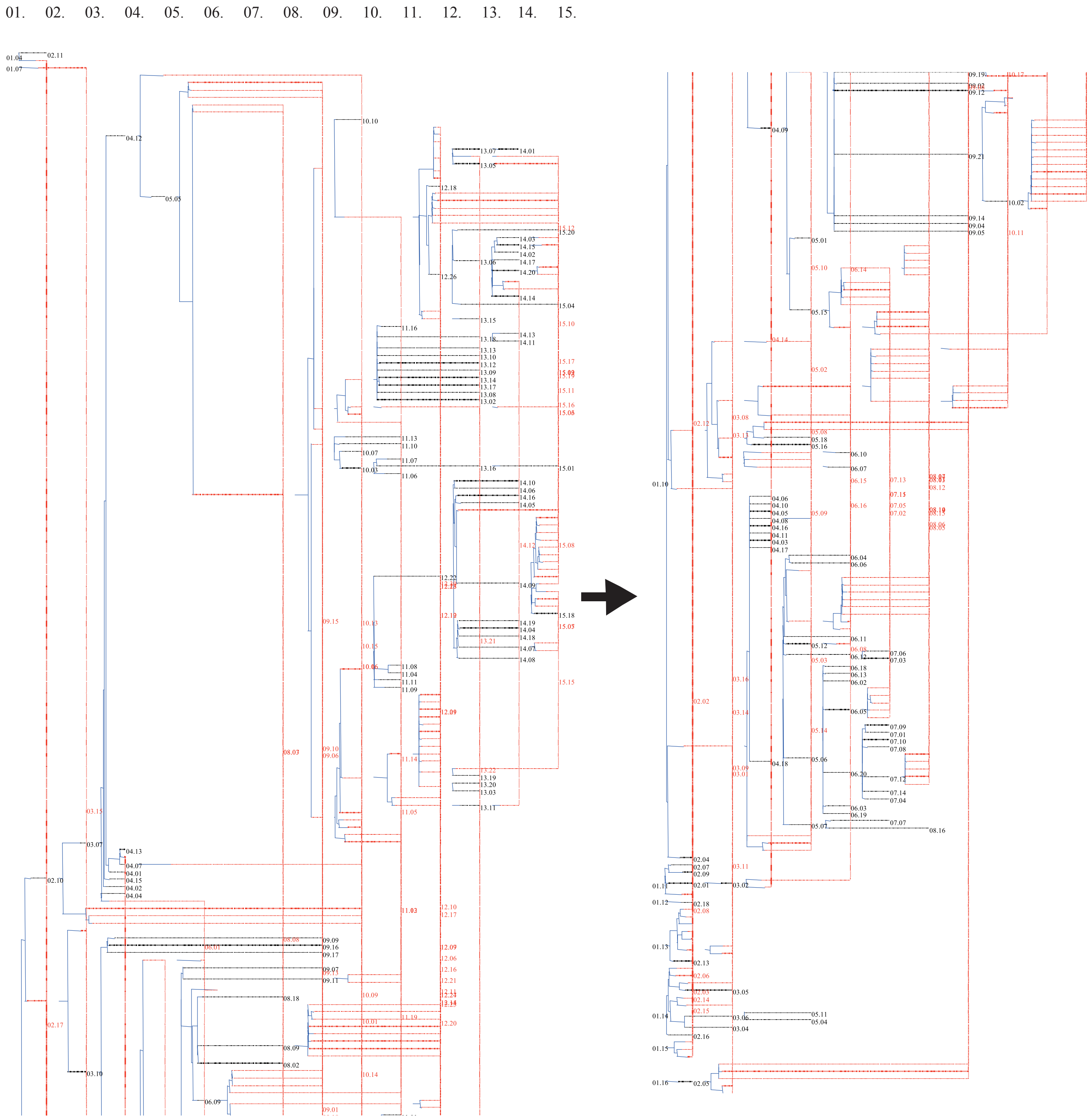

The evolutionary network of 273 PR genes from patient 1 constructed using Sliding MinPD method. The 15 sampling points are shown on the top. 01-15 correspond to A-O shown in Figure 1a and sequence numbers are the same as in additional file 3 (a). Solid lines indicate distances, while dotted lines extend the linking relationships. Dashed red lines are used to link recombinant sequences with their predicted donor sequences.
