# Supplementary material for: Inferring within-patient HIV-1 evolutionary dynamics under anti-HIV therapy using serial virus samples with vSPA
Source: BMC Bioinformatics. 2009 Oct 29;10:360. doi: 10.1186/1471-2105-10-360 (PMC2776027; doi:10.1186/1471-2105-10-360)
Supplement: Additional file 5 — The vSPA programs and four datasets analyzed in this study. The programs developed to carry out the vSPA algorithm and four datasets analyzed in this study and are included in this ZIP file. [file 1471-2105-10-360-S5.zip › vSPA/Instruction.pdf]

vSPA
viral Sequential Pathway Analysis

vSPA implements the method of Hasegawa et al 2009 (submitted) for inferring within-patient viral evolutionary process using serial viral data. The method calculates genetic distances between viral samples and uses the Pearson correlation coefficient to classify viral variants into different clusters and further identifies evolutionary pathways between viral clusters. A permutation test at the codon level is used to determine the threshold of the correlation coefficient for clustering viral sequences sampled at different time points.

Hasegawa N, Sugiura W, Shibata J, Matsuda M, Ren F and Tanaka H: Inferring within-patient HIV-1 evolutionary dynamics under anti-HIV therapy using serial virus samples with vSPA. 2009, submitted

The contents of vSPA program package

vSPA.pl
This is the main program for carrying out vSPA algorithm

acquired_mutation.pl
This program can be used for detecting mutations along the evolutionary pathways inferred by vSPA.pl

Library
This folder contains Perl and R programs needed for executing vSPA.pl and acquired_mutation.pl.

Example
This folder contains two data files of HIV-1 protease and reverse transcriptase genes from Patient 1 analyzed in our study

Output_example
This folder includes the result of vSPA inferred from dataset P1PR.fasta.

All programs are currently developed and tested on Mac OS X and Windows XP. For Windows users, some additional settings are requested (see Additional settings for Windows users)

■Installations

To run the vSPA programs, you need to install Perl and R package first. Perl can be found here: http://www.perl.com/download.csp and R package can be downloaded from: http://cran.r-project.org/. After installing the R package, you need to further install “ape” (Analysis of Phylogenetics and Evolution: http://ape.mpl.ird.fr) on R Console with

```
> install.packages (“ape”)
```

To view the evolutionary pathways inferred by vSPA, you also need to install an open-source graph visualization tool, Graphviz: http://www.graphviz.org/.

■Running the programs

Input file

The input file should be a set of already aligned sequences in fasta format with sequence IDs with the sampling time as a capital letter followed by an underscore followed by a sequence identifier as Arabic numerals. The sequences do not need to appear in order. See P1PR.fasta in "Example" folder for example.

To infer the evolutionary pathways from P1PR.fasta with 100 replicates of permutation, vSPA.pl is run with

```
> perl vSPA.pl Example/P1PR.fasta
```

■Options

-p number

Number of permutation replicates. Default: 100

-m model

The evolutionary model to be used; must be one of "raw", "N", "JC90", "K80", "F81", "F84", "BH84", "T92", "TN93", "GG95", "logdet", "paralin". Default: K80

-g number

A value for the gamma parameter which allows for sites in the sequence to evolve at different rates. Default: 0.5

Then, the "output" folder will be generated automatically which contains a folder and three files (see "Output_example" folder):

Matrix

This folder includes the following files

distance.txt

The genetic distance matrix inferred from all viral sequences is listed in this file. This matrix is generated for linking viral clusters based on their genetic distances.

timepoints.txt

All sampling time points are listed in this file.

Binary format matrices

These binary matrices are inferred from viral sequences sampled at different time points, in which '1' is for above the correlation coefficient threshold and '0' is for below the threshold.

For example, the binary format matrix inferred from viral sequences sampled at time point A is listed in A.txt.

These binary matrices are generated for clustering viral sequences.

vSPA.dot

This file is the main output of vSPA.pl. The inferred evolutionary pathways can be viewed using Graphviz.

cluster_seq.txt and cluster_link.txt

These two files are used as input to acquired_mutation.pl. For instance, for detecting acquired mutations along the pathways inferred

from P1PR, run

> perl acquired_mutation.pl Example/P1PR.fasta output/cluster_seq.txt output/ cluster_link.txt

cluster_dist.txt

The average genetic distance matrix inferred from all viral clusters is listed in this file.

cluster_range.txt

The range of genetic distances (minimum and maximum values) for each pair of clusters at different sampling time points is listed in this file.

Output of acquired_mutation.pl

The amino acid changes acquired along the pathways are collected with Position, AA (codon) and Frequency as follows

1. The terminal cluster of a pathway is listed with symbol '*' first followed by the ancestral cluster (it is also called "root cluster" in our article) and all descendent clusters belonging to this pathway.
2. The number of sequences in each cluster is shown in parentheses and the codon frequency is calculated based on this number.
3. Compared with the oldest ancestral cluster of a pathway, if an amino acid in its descendent clusters changed at high frequency (> 0.5), this amino acid will be identified as the acquired mutation and displayed with its position, codon composition and frequency under the descendent cluster name. The initial frequencies of these amino acids are also listed under the oldest ancestral cluster name.

■Additional settings for Windows users

1. Go to System Properties → Advanced → Environment Variables, then find "path" from User Variables and double click it to open Edit User Variable. Set Variable value as follows:

   ;C:\Program Files\Graphviz2.22\bin

2. To view the result of inferred pathways, start GVedit and open the vSPA.dot. Before clicking Run button, you need to find Settings button from the toolbar menu (next to the Run button) and set dot format for Layout Engine.
