# Supplementary figures and images for: Inferring within-patient HIV-1 evolutionary dynamics under anti-HIV therapy using serial virus samples with vSPA
Source: BMC Bioinformatics. 2009 Oct 29;10:360. doi: 10.1186/1471-2105-10-360 (PMC2776027; doi:10.1186/1471-2105-10-360)

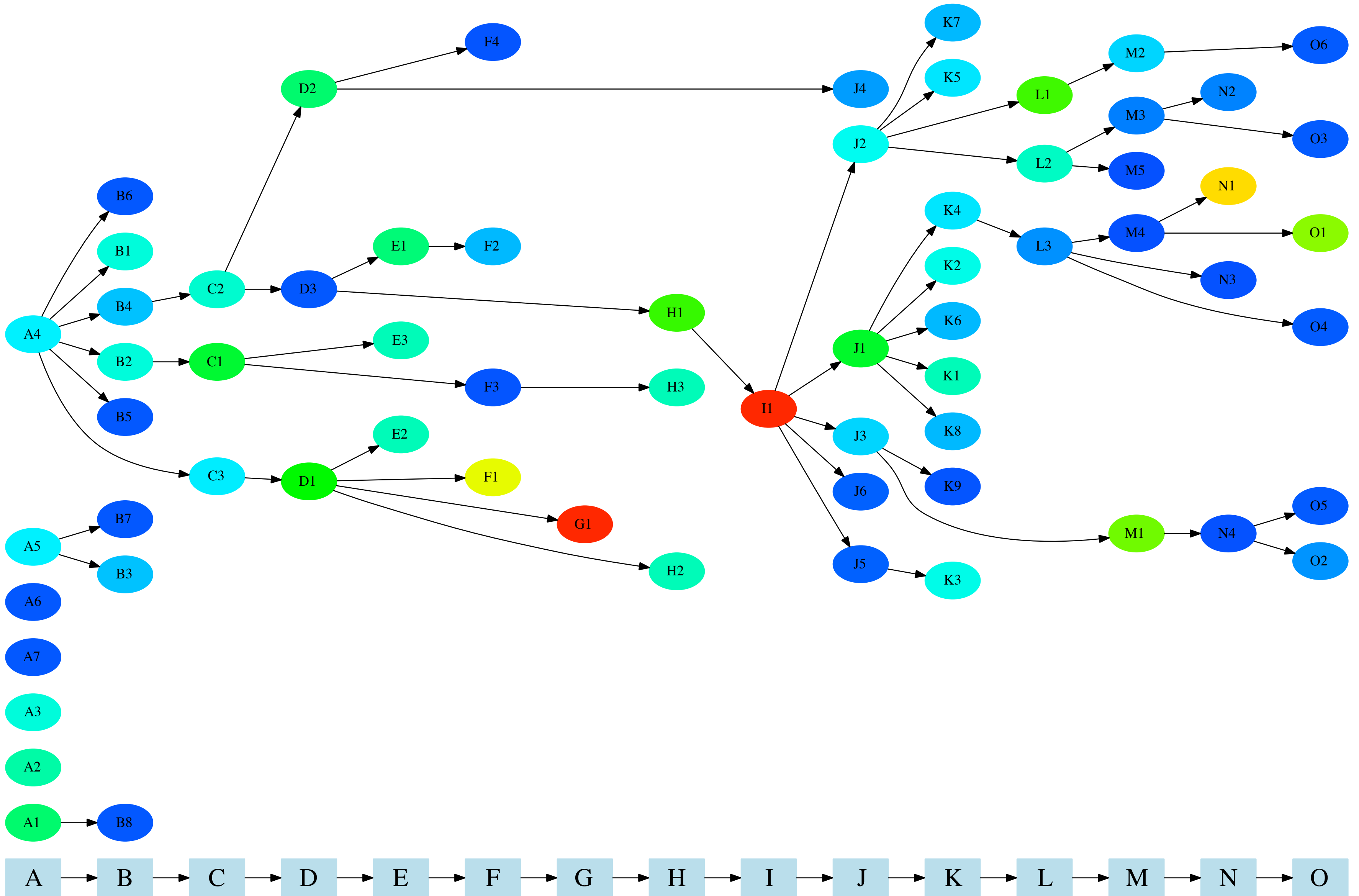

Supplement: Additional file 5 — The vSPA programs and four datasets analyzed in this study. The programs developed to carry out the vSPA algorithm and four datasets analyzed in this study and are included in this ZIP file. [file 1471-2105-10-360-S5.zip › vSPA/Output_example/vSPA.pdf]
